# Insights Into the Resistome of Bovine Clinical Mastitis Microbiome, a Key Factor in Disease Complication

**DOI:** 10.3389/fmicb.2020.00860

**Published:** 2020-06-03

**Authors:** M. Nazmul Hoque, Arif Istiaq, Rebecca A. Clement, Keylie M. Gibson, Otun Saha, Ovinu Kibria Islam, Ruhshan Ahmed Abir, Munawar Sultana, AMAM Zonaed Siddiki, Keith A. Crandall, M. Anwar Hossain

**Affiliations:** ^1^Department of Microbiology, University of Dhaka, Dhaka, Bangladesh; ^2^Department of Gynecology, Obstetrics and Reproductive Health, Faculty of Veterinary Medicine and Animal Science, Bangabandhu Sheikh Mujibur Rahman Agricultural University, Gazipur, Bangladesh; ^3^Department of Developmental Neurobiology, Graduate School of Medical Sciences, Kumamoto University, Kumamoto, Japan; ^4^Computational Biology Institute, Milken Institute School of Public Health, The George Washington University, Washington, DC, United States; ^5^Department of Microbiology, Jashore University of Science and Technology, Jashore, Bangladesh; ^6^Bio-Bio-1, Bioinformatics Research Foundation, Dhaka, Bangladesh; ^7^Department of Pathology and Parasitology, Chittagong Veterinary and Animal Sciences University, Chittagong, Bangladesh; ^8^Department of Biostatistics and Bioinformatics, Milken Institute School of Public Health, The George Washington University, Washington, DC, United States

**Keywords:** whole metagenome sequencing, clinical mastitis, microbiome, resistome, *in vitro* resistance assays

## Abstract

Bovine clinical mastitis (CM) is one of the most prevalent diseases caused by a wide range of resident microbes. The emergence of antimicrobial resistance in CM bacteria is well-known, however, the genomic resistance composition (the resistome) at the microbiome-level is not well characterized. In this study, we applied whole metagenome sequencing (WMS) to characterize the resistome of the CM microbiome, focusing on antibiotics and metals resistance, biofilm formation (BF), and quorum sensing (QS) along with *in vitro* resistance assays of six selected pathogens isolated from the same CM samples. The WMS generated an average of 21.13 million reads (post-processing) from 25 CM samples that mapped to 519 bacterial strains, of which 30.06% were previously unreported. We found a significant (*P* = 0.001) association between the resistomes and microbiome composition with no association with cattle breed, despite significant differences in microbiome diversity among breeds. The *in vitro* investigation determined that 76.2% of six selected pathogens considered “biofilm formers” actually formed biofilms and were also highly resistant to tetracycline, doxycycline, nalidixic acid, ampicillin, and chloramphenicol and remained sensitive to metals (Cr, Co, Ni, Cu, Zn) at varying concentrations. We also found bacterial flagellar movement and chemotaxis, regulation and cell signaling, and oxidative stress to be significantly associated with the pathophysiology of CM. Thus, identifying CM microbiomes, and analyzing their resistomes and genomic potentials will help improve the optimization of therapeutic schemes involving antibiotics and/or metals usage in the prevention and control of bovine CM.

## Introduction

Mastitis is the foremost production and major economic burden confronted by the global dairy industry (Reyes-Jara et al., 2016; Hoque et al., 2019). Bovine clinical mastitis (CM) milk is now considered to host a complex microbial community with great diversity (Oikonomou et al., 2014; Falentin et al., 2016; Hoque et al., 2019). The most frequently isolated pathogens are *Staphylococcus aureus, Escherichia coli, Klebsiella* spp., *Streptococcus* spp., *Mycoplasma* spp., *Enterobacter* spp., *Bacillus* spp., and *Corynebacterium* species (Abebe et al., 2016; Gao et al., 2017; Hoque et al., 2018). Therefore, the accurate identification of pathogens that cause CM enables appropriate choices for antimicrobial treatment and preventive mastitis management (Preethirani et al., 2015; Van Boeckel et al., 2015; Cheng et al., 2019). Over the past two decades, a wide range of phenotyping and genotyping methods have been implemented to study mastitis-causing bacteria (Preethirani et al., 2015; Gao et al., 2017; Hoque et al., 2018; Cheng et al., 2019). Although culture-based techniques are traditionally used to detect CM bacteria, these methods are time-consuming and have the inherent drawback of not being applicable to non-cultivable bacteria (Baron et al., 2018). Until recently, 16S rRNA partial gene sequencing remained the most commonly used genomic survey tool to study bovine mastitis microbiomes (Oikonomou et al., 2014; Falentin et al., 2016; Cremonesi et al., 2018). However, this technique has limitations because of polymerase chain reaction (PCR) bias, lower taxonomic resolution at the species level, and limiting information on gene abundance and functional profiling (Oniciuc et al., 2018). Shotgun whole metagenome sequencing (WMS), on the other hand, better characterizes the breadth of microbial diversity in a sample and successfully provides insight into the phylogenetic composition, species and/or strain, and functional diversity for a variety of biomes (Seth et al., 2014; Oniciuc et al., 2018). This WMS typically produces high complexity datasets with millions of short reads allowing extensive characterization of the microbiome in an ecological niche, profiling its functional attributes, and gradually becoming a cost-effective metagenomic approach (Seth et al., 2014; Oniciuc et al., 2018; Hoque et al., 2019).

Currently, antimicrobial treatment is indispensable to keeping bovine udder health, animal welfare, and economic aspects in balance (Preethirani et al., 2015; Krömker and Leimbach, 2017; Cheng et al., 2019). Therefore, dependence on antimicrobials has become a widespread phenomenon on dairy farms for mastitis management, prevention, and control programs. However, efficacy of antimicrobial therapy against bovine CM pathogens is low (Cheng et al., 2019), and the use of antibiotics is confined to selected severe CM cases only (Reyes-Jara et al., 2016; Cheng et al., 2019). The prevalence of antimicrobial resistance (AMR) in bovine CM pathogens has been investigated in numerous studies (Preethirani et al., 2015; Krömker and Leimbach, 2017; Cheng et al., 2019). The secretion of antimicrobial compounds by microbes is an ancient and effective method to improve the survival of microbes competing for space and nutrients with other microorganisms (D’Costa et al., 2011). The vast diversity of bacterial species in CM milk, many with short generation times and rampant horizontal gene transfer, permit the rapid accumulation of countless resistant variants at a relatively high evolutionary pace (D’Costa et al., 2011; Weller and Wu, 2015). However, resistance in CM bacteria typically goes unnoticed until a given species becomes of clinical interest, and the associated resistome is also suspected to be a source of newly emerging resistance genes in CM pathogens (Krömker and Leimbach, 2017; Cheng et al., 2019; Hoque et al., 2019; Zaheer et al., 2019). Bacteria residing in the bovine gastrointestinal tract and udder may become resistant to these antibiotics and, once released into the milk, they may take part in horizontal transfer of antibiotic resistance genes (ARGs) to other CM bacteria of contagious and environmental origin (Cheng et al., 2019; Zaheer et al., 2019). Furthermore, AMR is a global health concern in both human and veterinary medicine (Van Boeckel et al., 2015), and thus, monitoring the emergence of AMR strains is an essential component of bovine CM prevention and control strategies (Tomazi et al., 2018; Cheng et al., 2019). Therefore, finding an effective alternative strategy for the control of bovine mastitis is a challenge for dairy producers.

The antimicrobial properties of metals have been documented throughout the history of medicine and healthcare (Vaidya et al., 2017). Metal salts such as chromium (Cr), cobalt (Co), nickel (Ni), copper (Cu), and zinc (Zn) are effective in controlling bacterial transmission and infection risks (Vaidya et al., 2017). However, their uses are limited due to their toxicity and possible detrimental environmental effects in dairy industries particularly as therapeutic agents against bovine CM pathogens. Biofilm formation (BF) is an important virulence factor for mastitis causing bacteria and contributes to resistance to different classes of antimicrobials (Singh et al., 2017). Bacterial pathogens identified in this study showed a broad spectrum of antimicrobial (antibiotics, toxic metals) resistance, and possessed biofilm forming and quorum sensing (QS) abilities, which might be potential factors in hindering CM cures; thereby leading to the persistence of the disease, and the increased risk of transmission to non-infected dairy cows. However, genetic information about resistance or *in vitro* assays of resistance is not enough to understand the resistome when considered in isolation rather than in combination (Van Boeckel et al., 2015; Baron et al., 2018). Here we describe the resistance potentials in the CM microbiome of four major cattle breeds (Local Zebu, LZ; Red Chattogram Cattle, RCC; Sahiwal, SW; Crossbred Holstein Friesian; XHF) of Bangladesh using both metagenomic deep sequencing (WMS) and *in vitro* cultural approaches. We aim to investigate the influences of metabolic genomic potentials of the microbiomes in predicting and understanding the role of resistant potentials in the pathophysiology of bovine CM. We also test if cattle breeds or host genetics influence the milk microbiota composition and susceptibility to disease and resistance to bacterial infection (Cremonesi et al., 2018; Curone et al., 2018; Gonzalez-Recio et al., 2018).

## Materials and Methods

### Ethics Statement

The protocol for milk sample collection from lactating dairy cows was approved by the Animal Experimentation Ethical Review Committee (AEERC), Faculty of Biological Sciences, University of Dhaka under reference number 79/Bio. Scs., dated: 12-12-2019.

### Screening for Clinical Mastitis and Sampling

We screened 260 udder quarter milk samples collected from 260 CM affected cows belonging to 50 smallholding dairy farms in two geographical regions of Bangladesh (central region, CR = 160; southeastern region, SER = 100) (Supplementary Figure S1). The cows represented four different breeds, including local zebu (LZ), red Chattogram cattle (RCC), Sahiwal (SW), and crossbred Holstein Friesian (XHF) at their early stage of lactation (within 10–40 days post-calving). A screening test for CM was conducted using the California Mastitis Test (CMT^®^, Original Schalm reagent, ThechniVet, United States) (Hoque et al., 2015). Approximately 15–20 mL of milk from each cow was collected under aseptic conditions in a sterile falcon tube during morning milking (8.00–10.00 am) and kept on ice (at 4°C) for transport to the laboratory for subsequent processing.

### Metagenomic DNA Extraction and Sequencing

Genomic DNA (gDNA) from 25 randomly selected CM samples was extracted by an automated Maxwell 16 DNA extraction platform using blood DNA purification kits (Promega, United Kingdom) following previously described protocols (Hoque et al., 2019). DNA quantity and purity were determined with NanoDrop (ThermoFisher, United States) by measuring 260/280 absorbance ratios. Sequencing libraries were prepared with the Nextera XT DNA Library Preparation Kit (Head et al., 2014) and paired-end (2 × 150 bp) sequencing was performed on a NextSeq 500 machine (Illumina Inc., United States) at the George Washington University Genomics Core facility. Our metagenomic DNA yielded a total of 596.74 million reads with an average of 23.87 million (maximum = 39.75 million, minimum = 8.89 million) reads per sample (Supplementary Material) before cleaning.

### Sequence Reads Preprocessing

The resulting FASTQ files were concatenated and filtered through BBDuk (Hoque et al., 2019) (with options *k* = 21, mink = 6, ktrim = r, ftm = 5, qtrim = rl, trimq = 20, minlen = 30, overwrite = true) to remove Illumina adapters, known Illumina artifacts, and phiX before bioinformatics analyses. Any sequence below these thresholds or reads containing more than one “N” were discarded. On average, 21.13 million reads per sample (maximum = 36.89 million, minimum = 4.71 million) passed the quality control step (Supplementary Material).

### Microbiome Diversity and Community Analysis

The shotgun WMS data were analyzed using both mapping-based and assembly-based hybrid methods of PathoScope 2.0 (PS) (Hong et al., 2014) and MG-RAST (MR) (Glass et al., 2010), respectively. In PS analysis, a “target” genome library was constructed containing all bacterial sequences from the NCBI Database using the PathoLib module (Hong et al., 2014). The reads were then aligned against the target libraries using the very-sensitive Bowtie 2 algorithm (Langmead and Salzberg, 2012) and filtered to remove the reads aligned with the cattle genome (bosTau8) and human genome (hg38) as implemented in PathoMap (-very-sensitive-local -k 100–score-min L,20,1.0). Finally, the PathoID (Francis et al., 2013) module was applied to obtain accurate read counts for downstream analysis. In these samples, 17.20 million reads (4.3% of total reads) mapped to the target reference genome libraries after filtering for the cow and human genome (Supplementary Material). The raw sequences were simultaneously uploaded to the MR server (release 4.0), with proper embedded metadata, and were subjected to the quality filter containing dereplication and removal of host DNA by screening for taxonomic and functional assignment. Alpha diversity (diversity within samples) was estimated using the observed species, Chao1, ACE, Shannon, Simpson and Fisher diversity indices (Koh, 2018) for both PS and MR read assignments and counts. To visualize differences in bacterial diversity, a principal coordinate analysis (PCoA) was performed based on weighted-UniFrac distances (for PS data) through the Phyloseq R package, version 3.5.1 (McMurdie and Susan, 2013) and Bray-Curtis dissimilarity matrix (Beck et al., 2013) (for MR data). We also used OmicCircos, version 3.9 (Hu et al., 2014a), an R package based on python scripts, for circular visualization of both microbiome diversity and resistance to antibiotics and toxic compounds (RATC) functional groups found in MR data for our four targeted breeds of CM cows.

### *In vitro* Identification of Bacteria

Collected CM milk samples (*n* = 260) were subjected to selective isolation and identification of *S. aureus*, *E. coli*, *Klebsiella*, *Enterobacter*, *Shigella*, and *Bacillus* species according to previously described microbiological methods (Reyes-Jara et al., 2016; Gao et al., 2017; Hoque et al., 2018; Cheng et al., 2019). The pathogens were identified based on their colony morphology, hemolytic patterns on blood agar and Gram-staining (Cheng et al., 2019). Gram-positive bacteria were further confirmed based on their biochemical characteristics in indole, methyl red, Voges-Proskauer (VP), catalase, oxidase, urease and triple sugar iron (TSI) tests, and growth on mannitol salt agar. Gram-negative bacteria were confirmed based on the results of indole, methyl red, citrate (IMViC) tests and lactose fermentation on MacConkey agar (Preethirani et al., 2015; Gomes et al., 2016). Finally, all isolates were stored at −80°C for further genomic identification.

### PCR Amplification and Ribosomal (16S rRNA) Gene Sequencing

Genomic DNA of probable *S. aureus*, *E. coli*, *Klebsiella*, *Enterobacter*, *Shigella*, and *Bacillus* species was extracted from overnight cultures using the boiled method Queipo-Ortuño et al., 2008). The quantity and purity of the extracted DNA was determined as mentioned before. The 16S rRNA gene was amplified using universal primers 27F (5′-AGAGTTTGATCCTGGCTCAG-3′) and U1492R (5′-CTACGGCTACCTTGTTACGA-3′) (Masomian et al., 2016). Agarose gel electrophoresis (1.2% wt/vol) was used to verify the presence of PCR products. DNA sequencing was carried out at First Base Laboratories Sdn Bhd (Malaysia) using Applied Biosystems highest capacity-based genetic analyzer (ABI PRISM^®^ 377 DNA Sequencer) platforms with the BigDye^®^ Terminator v3.1 cycle sequencing kit chemistry.

### Phylogenetic Analysis of the Microbial Communities

Taxonomic abundance of the WMS data was determined by applying the “Best Hit Classification” option in the PS pipeline using the NCBI database as a reference, with the following settings: maximum e-value of 1 × 10^–30^, minimum identity of 95% for bacteria, and a minimum alignment length of 20 as the set parameters (Masomian et al., 2016). A midpoint rooted phylogenetic tree consisting of the top 200 abundant bacterial strains, identified through PS analysis from the WMS reads of the 25 CM samples with >90% taxonomic identity, was constructed using the maximum-likelihood method in Clustal W, version 2.1 (Larkin et al., 2007) and visualized using the interactive Tree Of Life (iTOL) (Letunic and Bork, 2011). Another phylogenetic tree, which was constructed using the same approach, focused on 40 strains corresponding to the six *in vitro* examined CM bacteria found in 260 CM samples with >90% taxonomic identity. Using Molecular Evolutionary Genetics Analysis (MEGA) version 7.0 for the larger datasets (Kumar et al., 2016), the 16S rRNA gene sequences, amplified from all individual bacterial isolates, were aligned with each other and with relevant reference sequences obtained from the NCBI Database using MEGA 7.0, and a maximum-likelihood tree was generated using these 16S rRNA gene sequences with the Tamura-Nei evolutionary model (Kumar et al., 2016). Nodal confidence in the resulting phylogenetic relationships was assessed using the bootstrap test (1000 replicates) (Pattengale et al., 2010).

### Antimicrobial Susceptibility Testing

The *in vitro* antibiogram profile of 221 CM isolates was determined using the disk diffusion method (Supplementary Figure S2) following the Clinical Laboratory Standards Institute (CLSI, 2017) guidelines. Antibiotics were selected for susceptibility testing corresponding to a panel of antimicrobial agents (Oxoid^TM^, Thermo Scientific, United Kingdom) of interest to the dairy industry and public health in Bangladesh. The selected groups of antibiotics were commonly used in treating CM by the dairy farmers and included penicillins (ampicillin, 10 μg/mL), tetracyclines (doxycycline, 30 μg/mL; tetracycline, 30 μg/mL), nitrofurans (nitrofurantoin, 300 μg/mL), quinolones (ciprofloxacin, 10 μg/mL; nalidixic acid, 30 μg/mL), cephalosporins (cefoxitin, 30 μg/mL), penems (imipenem, 10 μg/mL), phenols (chloramphenicol, 30 μg/mL), aminoglycosides (gentamycin, 10 μg/mL; vancomycin, 30 μg/mL), and macrolides (erythromycin, 15 μg/mL). Resistance was defined according to the clinical and laboratory standards institute (CLSI, 2017) with slight modifications (Preethirani et al., 2015; Cheng et al., 2019).

### Metal Susceptibility Testing

The antibacterial effect of heavy metals was evaluated *in vitro* for the isolated pathogens using the agar well diffusion method (Supplementary Figure S3) (Reyes-Jara et al., 2016; Vaidya et al., 2017). Five heavy metals such as copper (Cu), zinc (Zn), chromium (Cr), nickel (Ni), and cobalt (Co) were used as salts: CuSO_4_⋅5H_2_O, ZnSO_4_⋅7H_2_O, K_2_Cr_2_O_7_, NiCl_2_, and CoCl_2_⋅6H_2_O, respectively, to study the level of zone of inhibition (ZOI). Briefly, pure culture of the isolated pathogens from NA plates were sub-cultured into Mueller-Hinton agar (Oxoid^TM^, United Kingdom) plates, and respective agar was poured into sterile Petri dishes, which were then cooled. A total of 100 μl of cell suspension was pipetted and spread across the entire area of the agar using a sterile cotton swab. Two to five equal wells (7 mm diameter) were cut out of each agar plate using a sterile cork borer and stainless-steel needle. Varying concentrations of the metal solutions were prepared (2, 4, 8, 16, 32, 48, and 64 μg/mL) and 100 μl of the prepared metal ion solution was added to each of the wells. The plates were incubated at 37°C for 24 h to allow diffusion of the metal into the agar, and the antibacterial activity was determined by measuring the diameter of ZOI in mm (Cremonesi et al., 2018). After investigating the resistance profile of the isolates at different concentrations, the minimal inhibitory concentration (MIC) of the metals was determined using the tube dilution method, by gradually increasing or decreasing the heavy metal concentrations (Reyes-Jara et al., 2016). Finally, growth of bacterial colonies was observed and the concentration that showed no growth was considered as the minimum bactericidal concentration (MBC) (Reyes-Jara et al., 2016).

### Biofilm Assay and Microscopy

Microtiter plate assays were performed to screen for the BF ability of 80 randomly selected isolates using standard protocols (Schönborn et al., 2017; Singh et al., 2017; Vaidya et al., 2017). We quantified the absorbance of solubilized crystal violet (CV), in a plate reader at 600 nm using 30% acetic acid in water as the blank and TSB as the negative control. The solution was removed, and the absorbance measured at optical density-590 (OD590) (*n* = 3). To determine the BF ability of strains, cut-off optical density (ODc) was defined as three standard deviations above the mean OD of the negative control. Strains were classified as: non-biofilm formers, NBF (OD ≤ ODc); weak biofilm formers, WBF (ODc < OD ≤ 2 × ODc); moderate biofilm formers, MBF (2 × ODc < OD ≤ 4 × ODc), and strong biofilm formers, SBF (OD > 4 × ODc) (Tiwari et al., 2017; Vaidya et al., 2017). In this study, the ODc value was set as 0.045 and the mean OD of the negative control was 0.039 ± 0.002 (Vaidya et al., 2017). The biofilm surfaces were then visualized using 5% TSB as nutrient rich media and FilmTracer^TM^ LIVE/DEAD^®^ Biofilm Viability Kit as staining materials to observe the proportion of live or active cells (fluorescent green) under Olympus BX51 upright microscope (40× objective), and finally, images were collected using an Olympus DP73 camera through cellSens entry software (Olympus Corporation, Japan) and visualized using image J software (Schönborn et al., 2017). As a negative control, we used *E. coli* DH5 alpha for all the *in vitro* resistome (antimicrobial and metal susceptibility tests and biofilm assays) analysis tests.

### Microbial Functional Analysis

Metagenomic functional composition was based on the gene families from different levels of the SEED module and the Kyoto Encyclopedia of Genes and Genomes (KEGG) database (Kanehisa et al., 2019), using the MR pipeline (Glass et al., 2010). We observed significant differences (Kruskal–Wallis test, *P* = 0.001) in the relative abundance of genes coding for RATC and microbial functional genomic potentials in four cattle breeds.

### Statistical Analyses

The characteristics of the cow breeds with CM were compared using a non–parametric Kruskal–Wallis test for quantitative variables (Cremonesi et al., 2018). The Shapiro-Wilk test was used to check normality of the data, and the non–parametric Kruskal–Wallis rank sum test was used to evaluate differences in the relative abundance of bacterial taxa at the strain level according to breed groups (Cremonesi et al., 2018; Curone et al., 2018; Gonzalez-Recio et al., 2018). The statistical analyses for the MR data were initially performed by embedded calls to statistical tests in the pipeline and validated further using IBM SPSS (SPSS, Version 23.0, IBM Corp., NY, United States) using the above-mentioned tests. For the functional abundance profiling, the statistical (Kruskal–Wallis test and Pearson correlation) tests were applied at different KEGG and SEED subsystem levels in MR pipeline generated data (Glass et al., 2010). To evaluate the significant relationships between identified bacterial species and the study region, we used the two-sample proportions test using SPSS. Results were considered statistically significant when *P* < 0.05 and highly significant when *P* < 0.01. Mean values were used to compare the antimicrobial efficacy results of the tested antibiotics and heavy metals at varying concentrations. Standard error means were calculated to analyze the distribution of the data from the mean value, and confidence intervals of 95% were calculated for the MIC and MBC tests results to plot error bars (Schönborn et al., 2017; Vaidya et al., 2017). We also performed Pearson correlation tests to assess the relationships between the taxonomic abundance of the pathogens and AMR, both for cultural and metagenomic data. A *post hoc* Bonferroni test was used to compare the biofilm OD600 mean values (Schönborn et al., 2017; Vaidya et al., 2017).

## Results

To decipher the resistance potentials in bovine CM microbiomes, we used both *in silico* (WMS, 16S rRNA gene sequencing) and *in vitro* (culture base) approaches. The present WMS investigation leads to the direct and comprehensive evaluation of resistance to antibiotics and toxic compounds (RATC), BF and QS genes in 25 CM milk samples. Furthermore, *in vitro* AMR profiling of six CM causing bacteria (*S. aureus*, *E. coli*, *Klebsiella*, *Enterobacter*, *Bacillus*, and *Shigella*) isolated from 260 CM milk samples was carried out using 12 commonly used antibiotics (ampicillin, doxycycline, tetracycline, nitrofurantoin, ciprofloxacin, nalidixic acid, cefoxitin, imipenem, chloramphenicol, gentamycin, erythromycin, and vancomycin), and five toxic metals (copper, zinc, chromium, nickel, and cobalt). Moreover, we also demonstrated some functional metabolic potentials of CM microbiomes found to be associated with mammary gland pathogenesis.

### Sequence Analysis

The WMS of 25 CM milk samples generated approximately 600 million reads, ranging from 8.86 to 39.75 million per sample. An average of 21.13 million reads per sample (maximum = 36.89 million, minimum = 4.71 million) passed the quality control step (Supplementary Material). We analyzed the sequencing reads simultaneously using two bioinformatics pipelines, PathoScope 2.0 (PS) and MG-RAST (MR).

### Microbiome Diversity and Composition in CM

**FIGURE 1:**
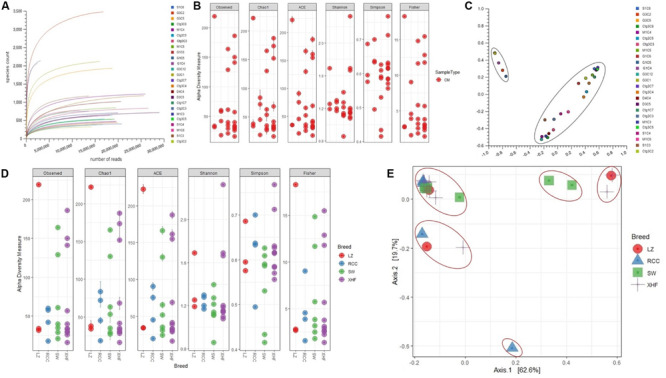
Bovine clinical mastitis (CM) milk microbiome diversity. **(A)** Rarefaction curves showing the influence of sequencing depth (number of reads per sample, *x*-axis) on species richness (*y*-axis) in CM milk samples. The rarefaction curves representing the number of species per sample indicated that the sequencing depth was sufficient enough to fully capture the microbial diversity as existed. **(B)** Alpha diversity measured using the observed species, Chao 1, ACE and Shannon diversity indices through PathoScope (PS) analysis. The observed species richness (*P*_Observed_ = 0.511), Chao1 (*P*_Chao__1_ = 0.081), ACE (*P*_ACE_ = 0.121), Shannon (*P*_Shannon_ = 0.401), Simpson (*P*_Simpson_ = 0.011) and Fisher (*P*_Fisher_ = 0.014) diversity analyses revealed that microbiome diversity did not vary among the CM samples. **(C)** Beta diversity (Principal coordinate analysis; PCoA) measured on the Bray-Curtis distance method using MG-RAST tool for CM causing microbial communities (genus-level) shows that most of the CM samples clustered together (black circle), indicating no significant diversity differences. **(D)** Alpha diversity measured using species richness (*P*_Observed_ = 0.011), Chao1 (*P*_Chao__1_ = 0.001), ACE (*P*_ACE_ = 0.021), Shannon (*P*_Shannon_ = 0.001), Simpson (*P*_Simpson_ = 0.009) and Fisher (*P*_Fisher_ = 0.023) diversity matrices on PS data showed significant differences (Kruskal–Wallis test, *P* = 0.002) in microbial diversity across the four cow breeds (Local Zebu cows, LZ; Red Chattogram cows, RCC; Sahiwal, SW; Holstein Friesian cross, XHF). **(E)** PCoA plot based on weighted-UniFrac distance method at strain-level microbiome signature of four breeds of cows reveals that the CM samples appear more distantly (red circles) indicating significant group differences (*P* = 0.001). These differences in the microbiome signature associated with CM across the four breeds could be explained by a large percentage of variation in the first (62.6%) and second (19.7%) axes.

We investigated the strain-level microbial community and relative abundances in 25 CM milk samples [previously published 14 samples (Hoque et al., 2019) and 11 new samples] through WMS. The reads generated from WMS were mapped to 391 genera and 519 strains of bacteria through MR and PS analyses, respectively (Supplementary Material). The rarefaction curves based on observed species richness reached a plateau after, on average, 23.87 million reads (Figure 1A and Supplementary Material), suggesting that the depth of coverage for most samples was sufficient to capture the entire microbial diversity within each sample. We did not, however, find any significant differences in the alpha (observed species, Chao1, ACE, Shannon, Simpson and Fisher diversity estimates) and beta (based on Bray-Curtis dissimilarity matrix) diversities among the microbial communities across the 25 CM samples (Figures 1B,C, respectively). However, significant diversity (alpha and beta) differences were observed among the CM microbiome communities across the four cattle breeds (LZ, RCC, SW, XHF) regardless of the method (i.e., either PS or MR) used to tabulate microbial abundances (PS; *P* = 0.005, MR; *P* = 0.001, Kruskal–Wallis test). In addition, this breed’s specific diversity difference remained evident in the microbial ecosystem of XHF cows associated CM milk samples (Figures 1D,E, respectively). The PCoA analysis also showed significant microbial disparity (*P* = 0.001) among the microbiome of four dairy breeds (Figure 1E).

**FIGURE 2:**
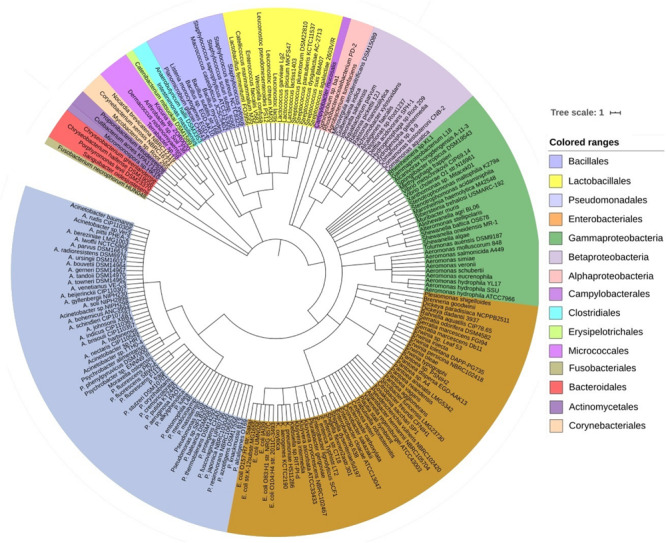
The strain-level taxonomic profile of microbiota associated with bovine clinical mastitis (CM). Taxonomic dendrogram showing the top bacterial microbiome of bovine CM milk. Color ranges identify different strains within the tree. Taxonomic dendrogram in the midpoint rooted phylogenetic tree was generated with the top 200 abundant unique strains of bacteria in CM milk metagenome based on the maximum likelihood method in Clustal W and displayed with iTOL (interactive Tree Of Life). Each node represents a single strain shared among more than 50% of the samples at a relative abundance of >0.0006% of the total bacterial community. Strains and/or species are color-coded by different order of bacteria present in >80% of samples. The strains in the phylogenetic tree are also available in Supplementary Material.

**FIGURE 3:**
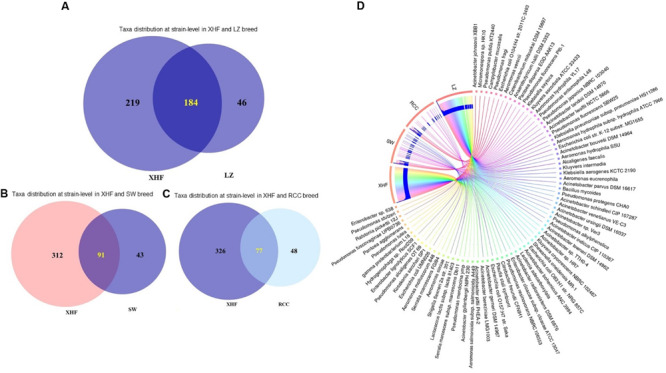
Strain-level bovine CM microbiome diversity in four different breeds (Local Zebu, LZ; Red Chattogram Cattle, RCC; Sahiwal, SW; Crossbred Holstein Friesian, XHF) of cows through PathoScope analysis. **(A)** Venn diagrams representing the core unique and shared microbiomes of bovine clinical mastitis (CM) in XHF and LZ breeds while **(B)** and **(C)** Venn diagrams showing the unique and shared bacterial strains in XHF and SW and XHF and RCC breeds, respectively. Microbiome sharing between the conditions are indicated by yellow color. **(D)** The circular plot illustrates the relative abundance of the top 75 CM causing bacterial strains in CM milk samples obtained from XHF, LZ, SW, and RCC dairy breeds. Taxa in the respective breed of cows are represented by different colored ribbons, and the inner blue bars indicate their respective relative abundances. The XHF cows had the highest number of microbial strains followed by LZ, SW, and RCC. This breed specific association revealed that 45.66, 22.58, and 19.11% of the detected bacterial strains in CM milk collected from LZ, SW, and RCC cows, respectively, were also seen in the CM milk microbiome of XHF cows. The relative abundance bacterial strains in four breeds is also available in Supplementary Material.

The predominant bacterial phyla we found associated with CM were Proteobacteria, Bacteroidetes, Firmicutes, Actinobacteria, and Fusobacteria (contributing to >95.0% of the total sequences, Kruskal–Wallis test, *P* = 0.001) in the MR analysis. The strain-level signature of the microbiome demonstrated that most of the species identified in each CM sample were represented by multiple strains (Supplementary Material), and of the detected bacterial strains, we identified the top 200 strains according to their relative abundance (Figure 2). The CM associated microbiome was dominated by 29 different strains of *Pseudomonas*, while *Acinetobacter*, *Streptococcus*, *Lactobacillus*, *Corynebacterium*, *Staphylococcus*, and *Enterococcus* were represented by 27, 27, 18, 17, 15, and 10 different strains, respectively (Figure 2 and Supplementary Material). Thus, among the identified bacterial strains, *A. johnsonii* XBB1 had the highest relative abundance (38.9%) followed by *Micromonospora* sp. HK10 (17.6%). Other bacterial strains with high relative abundance were *Campylobacter mucosalis* (8.7%), *P. putida* KT2440 (7.7%), *Anaerobutyricum hallii* DSM 3353 (6.3%), *P. fragi* (3.2%), *Catenibacterium mitsuokai* DSM 15897 (3.0%), *E. coli* O104:H4 str. 2011C-3493 (2.0%), *A. veronii* (1.2%), *Pantoea dispersa* EGD-AAK13 (1.1%), *P. fluorescens* Pf0-1 (0.8%), *K. oxytoca* (0.7%), and *P. entomophila* L48 (0.5%). The remaining strains had a relatively lower abundance (<0.5%) (Supplementary Material). According to the cattle breeds, the XHF cows had the highest number of microbial strains (*n* = 403) followed by LZ cows (*n* = 230), and SW cows (*n* = 134) and RCC (*n* = 125) (Figures 3A–C, and Supplementary Material). The breed specific association revealed that 45.7, 22.6, and 19.1% of the detected bacterial strains in CM milk samples of LZ, SW, and RCC cows, respectively, were also found in the CM microbiome of XHF cows (Figure 3D and Supplementary Material).

Simultaneously, through *in vitro* cultural analysis, a total of 452 isolates that belonged to six bacterial (*S. aureus*, *E. coli*, *Klebsiella*, *Enterobacter*, *Bacillus*, and *Shigella*) species were identified in 260 CM samples (including 25 WMS CM samples) collected from central (CR = 160) and southeastern (SER = 100) regions of Bangladesh (Supplementary Figure S1). The overall prevalence of *S. aureus*, *E. coli*, *Klebsiella*, *Enterobacter*, *Bacillus*, and *Shigella* species were 23.5, 18.5, 19.2, 12.3, 9.2, and 17.3% in CM samples, respectively (Supplementary Table S1). We found significant differences in the prevalence of these species (*P* = 0.01) when analyzing the distribution of these pathogens according to the origin of the samples (SER and CR) (Supplementary Figure S4). The culture-based findings of the current study identified *S. aureus* as the chief etiology of bovine CM in Bangladesh, while *Shigella* species remained the least frequently detected CM pathogen—which corroborates the results of WMS-based taxonomic identification (Supplementary Figure S5).

### Resistome Composition of CM Associated Microbiome

**FIGURE 4:**
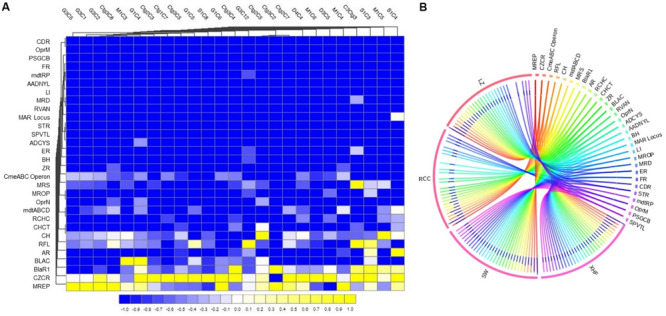
Projection of the resistance to antibiotic and toxic compounds (RATC) genes in bovine clinical mastitis (CM) pathogens. **(A)** Heatmap showing the hierarchical clustering of 30 different RATC genes detected in CM associated microbiomes of 25 CM milk samples as measured at level-3 of SEED subsystems in MG-RAST pipeline. The relative abundance of these genes significantly correlated (Pearson correlation, *P* = 0.002) with the relative abundance of the bacterial taxa found in these samples. The color bar at the bottom represents the relative abundance of putative genes and expressed as a value between –1 (low abundance) and 1 (high abundance). The yellow color indicates the more abundant patterns, while blue cells for less abundant RATC gene in that particular sample. The genes coding for MREP (multidrug resistance efflux pumps), CZCR (cobalt-zinc-cadmium resistance), BlaR (BlaR1 family regulatory sensor-transducer disambiguation); BLAC (beta-lactamase resistance), AR (arsenic resistance), RFL (resistance to fluoroquinolones), CH (copper homeostasis), CmeABC Operon (multidrug efflux pump in *Campylobacter jejuni*) had higher relative abundances than other RATC groups found in these CM samples. **(B)** The circular plot illustrates the diversity and relative abundance of the RATC genes detected among the microbiomes of the four different breeds (Local Zebu, LZ; Red Chattogram Cattle, RCC; Sahiwal, SW; Crossbred Holstein Friesian, XHF) of cows through SEED subsystems analysis. We found no significant correlation between the resistome and microbiome diversity in different breeds (*P* = 0.692). The association of the RATC genes according to breeds is shown by different colored ribbons and the relative abundances these genes are represented by inner blue colored bars. Part of the RATC functional groups are shared among microbes of the four breeds (XHF, LZ, SW, and RCC), and some are effectively undetected in the microbiomes of the other breeds. Abbreviations: CH, copper homeostasis; CHCT, copper homeostasis: copper tolerance; RCHC, resistance to chromium compounds; mdtABCD, the mdtABCD multidrug resistance cluster; OprN, mexe-mexf-oprn multidrug efflux system; MROP, mercury resistance to operon; MRS, methicillin resistance in *Staphylococci*; ZR, zinc resistance; BH, bile hydrolysis; ER, erythromycin resistance; ADCYS, adaptation to d-cysteine; SPVTL, *Streptococcus pneumoniae* vancomycin tolerance locus; STR, Streptothricin resistance; MAR Locus, multiple antibiotic resistance to locus; RVAN, resistance to vancomycin; MRD, mercuric reductase; LI, lysozyme inhibitors; AADNYL, aminoglycoside adenylyltransferases; mdtRP, multidrug resistance operon mdtRP of *Bacillus*; FR, Fosfomycin resistance; PSGCB, polymyxin synthetase gene cluster in *Bacillus*; OprM, mexA-mexB-oprm multidrug efflux system; CDR, cadmium resistance. Additional information is also available in Supplementary Material.

For analyses of the resistome composition in CM microbiomes, the SEED module of the MR pipeline provided a comprehensive picture. Using SEED, 147,040 reads aligned to 30 resistance to antibiotics and toxic compounds (RATC), and 10 BF and quorum sensing (BF-QS) functional groups across the CM samples, with different abundances (Supplementary Material). The RATC genes were classified into two unique groups, 19 antibiotic resistance and 11 toxic metal resistance groups (Figure 4 and Supplementary Material). This WMS analysis showed a significant association (Pearson correlation, *P* = 0.001; Non-parametric Spearman’s Correlation, *P* = 0.003) between the number of reads aligned to bacterial genomes and the number of reads mapped to RATC genes (Supplementary Material). Among the RATC functional groups, multidrug resistance to efflux pumps (MREP, 28.6%), *Cme*ABC operon (8.9%), resistance to fluoroquinolones (RFL, 6.2%), *mdt*ABCD cluster (5.5%), methicillin resistance in *Staphylococci* (MRS, 3.8%), *Bla*R1 regulatory family (*Bla*R1, 3.4%), *Mex*E-*Mex*F-*Opr*N (2.4%), and beta-lactamase resistance (BLAC, 2.2%) were the dominating antibiotic resistance genes (ARGs) found in CM milk microbiomes (Figure 4A and Supplementary Material). In addition to ARGs, the WMS analysis also detected a number of metal and toxic metals resistant genes in CM microbiomes. Among them, cobalt-zinc-cadmium resistance (CZCR, 19.3%), copper homeostasis (CH, 9.6%), arsenic resistance (AR, 2.9%), copper homeostasis: copper tolerance (CHCT, 2.3%), and resistance to chromium compounds (RCHC, 1.4%) were the predominate resistant genes (Figure 4A and Supplementary Material). Although the relative abundance of these RATC genes varied among the microbiomes of the four breeds (LZ, RCC, SW, and XHF), their resistome composition did not vary significantly (*P* = 0.692) by taxonomic diversity of respective breeds (Figure 4B and Supplementary Material).

**FIGURE 5:**
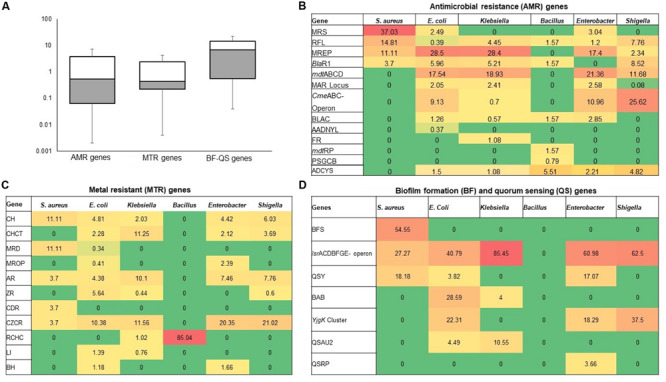
Heatmap comparison of antibiotics, metals, biofilm formation and quorum sensing genes found in the metagenome sequences (WMS) of six CM causing bacteria through SEED subsystems analysis in MG-RAST pipeline. **(A)** Diversity and relative abundance of the antimicrobial resistance (AMR), metal resistance (MTR), and biofilm formation (BF) and quorum sensing (QS) genes varied significantly (Kruskal–Wallis test, *P* = 0.029) among the study bacteria. **(B)** Relative abundance of AMR genes, **(C)** Relative abundance of MTR genes **(D)** Relative abundance of BF-QS genes. Values are colored in shades of green to yellow to red, indicating low (absent), medium and high abundance, respectively. Abbreviations: MRS, methicillin resistance in *Staphylococci*; RFL, resistance to fluoroquinolones; MREP, multidrug resistance to efflux pumps; BlaR, BlaR1 family regulatory sensor-transducer disambiguation; mdtABCD, the mdtABCD multidrug resistance cluster; MAR Locus, multiple antibiotic resistance; CmeABC Operon, Multidrug efflux pump in *Campylobacter jejuni*; BLAC, beta-lactamase resistance; AADNYL, aminoglycoside adenylyltransferases (Gentamycin resistance); FR, Fosfomycin resistance; mdtRP, multidrug resistance operon mdtRP of *Bacillus*; PSGCB, polymyxin synthetase gene cluster in *Bacillus*; BFS, biofilm formation in *Staphylococcus*, lsrACDBFGE operon, autoinducer 2 (AI-2) transport and processing; QSY, quorum sensing in *Yersinia*; BAB, biofilm adhesion biosynthesis; *Yjg*K cluster, protein *Yjg*K cluster linked to biofilm formation; QSAU2, quorum sensing: autoinducer-2 synthesis; QSRP, quorum sensing regulation in *Pseudomonas*; CH, copper homeostasis; CHCT, copper homeostasis: copper tolerance; MRD, mercuric reductase; MROP, mercury resistance to operon; AR, arsenic resistance; ZR, zinc resistance; CDR, cadmium resistance; CZCR, cobalt-zinc-cadmium resistance; ADCYS, adaptation to d-cysteine; RCHC, resistance to chromium compounds; LI, lysozyme inhibitors; BH, bile hydrolysis. More details about these genes can be found in the text and Supplementary Material.

The resistance potentials of RATC functional groups also varied significantly (*P* = 0.027) in six *in vitro* selected CM pathogens isolated and identified from different sources of CM samples (breed and study areas) under almost the same farming management system (Figure 5A and Supplementary Material). Among the RATC groups, the predominant ARGs found were as follows; MRS (*S. aureus*, 37.0%), RFL (*S. aureus*, 14.8%; *Shigella*, 7.8%), MREP (*E. coli*, 28.5%; *Klebsiella*, 28.4%), *Bla*R1 (*E. coli*, 6.0%; *Shigella*, 8.5%), *mdt*ABCD cluster (*E. coli*, 17.5%; *Klebsiella*,18.9%; *Enterobacter*, 21.4%; *Shigella*, 11.7%), multiple antibiotic resistance (MAR) Locus (*E. coli*, 2.4%; *Enterobacter*, 2.6%), *Cme*ABC operon (*E. coli*, 9.1%; *Enterobacter*, 11.0%; *Shigella*, 25.6%), and adaptation to d-cysteine, ADCYS (*Bacillus*, 5.5%) (Figure 5B). Conversely, genes encoding CH in *S. aureus* (11.1%), *E. coli* (4.8%), *Enterobacter* (4.4%), and *Shigella* (6.0%), CHCT in *Klebsiella* (11.2%) and *Shigella* (3.7%), mercuric reductase (MRD) in *S. aureus* (11.1%), mercury resistance to operon (MROP) in *Enterobacter* (2.4%), AR in *S. aureus* (3.7%), *E. coli* (4.4%), *Klebsiella* (10.1%), *Enterobacter* (7.5%) and *Shigella* (7.8%), ZR in *E. coli* (5.6%), cadmium resistance (CDR) in *S. aureus* (3.7%), CZCR in *S. aureus* (3.7%), *E. coli* (10.4%), *Klebsiella* (11.6%), *Enterobacter* (20.3%) and *Shigella* (21.0%), and RCHC in *Bacillus* (85.0%), were the most abundant toxic metals resistant RATC functional groups among the six selected pathogens (Figure 5C). Assessment of the BF-QS ability of the CM microbiomes revealed that autoinducer 2 (AI-2) transport and processing (*lsr*ACDBFGE operon, 33.7%), biofilm adhesion biosynthesis (BAB, 24.2%), protein *Yjg*K cluster linked to BF (*Yjg*K cluster, 15.5%), QS: autoinducer-2 synthesis (QSAU2, 9.4%) were the most abundant genes among CM associated pathogens (Supplementary Material). However, by comparing the association of these BF-QS genes among the six selected bacterial pathogens, we found significant variation (*P* = 0.017) in their diversity, composition, and relative abundances (Figure 5D and Supplementary Material).

**FIGURE 6:**
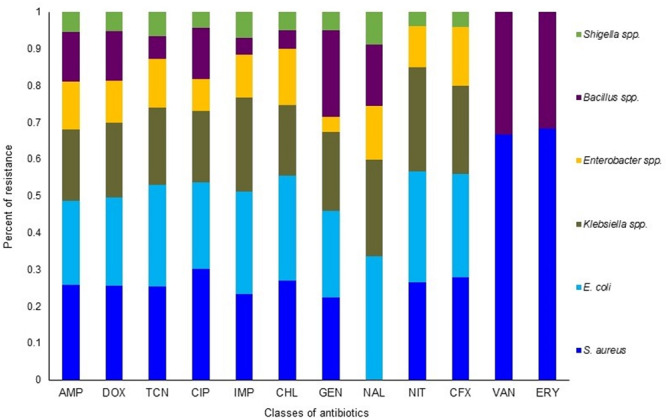
Antibiotic resistance pattern of bovine clinical mastitis pathogens by disk diffusion method. The antimicrobial resistance (AMR) patterns of the six bacteria obtained from 221 CM isolates (*S. aureus*, 56; *E. coli*, 54; *Klebsiella* spp., 42; *Enterobacter* spp., 26; *Bacillus* spp., 31; *Shigella* spp., 12) for twelve commonly used antibiotics from nine different groups/classes. Abbreviations: AMP, Ampicillin; DOX, Doxycycline; TCN, Tetracycline; CIP, Ciprofloxacin; IMP, Imipenem; CHL, Chloramphenicol; GEN, Gentamycin; NAL, Nalidixic acid; NIT, Nitrofurantoin; CFX, Cefoxitin; VAN, Vancomycin; ERY, Erythromycin. More details about AMR profiles can be found in the text and in Table 1.

The *in vitro* antibiogram profiling of 221 individual isolates of the six bacteria, revealed that *S. aureus* isolates had the highest resistance to doxycycline, ampicillin, tetracycline, and erythromycin (73.0–88.0%) and moderate resistance to chloramphenicol, ciprofloxacin, and nitrofurantoin (50.0–58.0%) (Figure 6 and Table 1). The isolates of another Gram-positive bacterium (*Bacillus*) demonstrated the highest resistance against doxycycline, ampicillin, nalidixic acid, and erythromycin (60.0–84.0%). However, *E. coli* isolates exhibited the highest resistance against tetracycline, doxycycline, nalidixic acid, and ampicillin (77.0–93.0%) and moderate resistance to chloramphenicol, nitrofurantoin, gentamicin, and ciprofloxacin (40.0–63.0%). The isolates of *Klebsiella*, *Enterobacter*, and *Shigella* displayed the highest resistance to doxycycline, nalidixic acid, tetracycline, and ampicillin (70.0–100.0%) and moderate resistance to ciprofloxacin, gentamicin, nitrofurantoin, and chloramphenicol (30.0–70.0%). In this study, imipenem and cefoxitin remained as the most sensitive antibiotics against four Gram-negative bacterial (*E. coli*, *Klebsiella*, *Enterobacter*, and *Shigella*) species, while the two Gram-positive (*S. aureus* and *Bacillus*) species were mostly sensitive to imipenem, cefoxitin, and vancomycin (Figure 6 and Table 1). Taken together, the antibiogram profile revealed that all of the selected CM pathogens are becoming multidrug resistant (MDR, resistant to ≥3 antibiotics) and the highest resistance was found against tetracyclines (tetracycline and doxycycline), followed by quinolones (nalidixic acid), and penicillin (ampicillin) groups of antibiotics (Figure 6 and Table 1).

**TABLE 1:** Antibiotic resistance pattern of bacteria [*n* (%) of isolates] associated with bovine clinical mastitis (CM).

Antibiotic	Content per disk	Breakpoint to declare resistance (≤)	*S. aureus* (*n* = 56)	*E. coli* (*n* = 54)	*Klebsiella* spp. (*n* = 42)	*Enterobacter* spp. (*n* = 26)	*Bacillus* spp. (*n* = 31)	*Shigella* spp. (*n* = 12)
AMP	10 μg	28 mm	48 (85.71)	42 (77.78)	36 (85.71)	24 (92.30)	25 (80.64)	10 (83.33)
DOX	30 μg	23 mm	49 (87.50)	46 (85.18)	39 (92.86)	22 (84.61)	26 (83.87)	10 (83.33)
TCN	30 μg	23 mm	46 (82.14)	50 (92.59)	38 (90.48)	24 (92.30)	11 (35.48)	12 (100)
CIP	10 μg	20 mm	28 (50.0)	22 (40.74)	18 (42.86)	8 (30.77)	13 (41.94)	4 (33.33)
IMP	10 μg	22 mm	10 (17.86)	12 (22.22)	11 (26.19)	5 (19.23)	2 (6.45)	3 (25.0)
CHL	30 μg	12 mm	32 (57.14)	34 (62.96)	23 (54.76)	18 (69.23)	6 (19.35)	6 (50.00)
GEN	10 μg	12 mm	22 (39.28)	23 (42.60)	21 (50.0)	4 (15.38)	23 (74.19)	5 (41.67)
NAL	30 μg	16 mm	ND	46 (85.18)	36 (85.71)	20 (76.92)	23 (74.19)	12 (100)
NIT	10 μg	64 mm	28 (50.0)	32 (59.25)	30 (71.42)	12 (46.15)	ND	4 (33.33)
CFX	30 μg	24 mm	14 (25.0)	14 (25.0)	12 (28.57)	8 (30.77)	ND	2 (16.67)
VAN	30 μg	20 mm	12 (21.42)	ND	ND	ND	6 (19.35)	ND
ERY	15 μg	20 mm	41 (73.21)	ND	ND	ND	19 (61.29)	ND

**n*, total number of isolates tested; ND, not done; AMP, ampicillin; DOX, doxycycline; TCN, tetracycline; CIP, ciprofloxacin; IMP, imipenem; CHL, chloramphenicol; GEN, gentamycin; NAL, nalidixic acid; NIT, nitrofurantoin; CFX, cefoxitin; VAN, vancomycin; ERY, erythromycin.*

**FIGURE 7:**
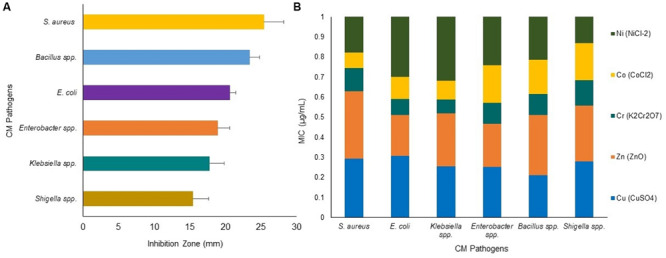
Antibacterial activity of heavy metals: Cu (CuSO_4_), Zn (ZnO), Cr (K_2_Cr_2_O_7_), Co (CoCl_2_) and Ni (NiCl_2_) against bovine CM pathogens. **(A)** Zone of inhibition (ZOI, mm) for six CM causing bacteria, each bar representing the mean values (values given horizontal axis of the bars, mm) and standard deviation error bar (SD error bar) for each bacterium. **(B)** Minimal inhibitory concentration (MIC) (expressed as μg/mL) of the tested metals against representative genera/species as determined by agar well diffusion method.

The use of toxic metals in soluble forms as an alternative to prevent bovine CM appears to be a novel promising idea supported by several earlier studies (Reyes-Jara et al., 2016; Vaidya et al., 2017). Zones of inhibition (ZOI) assays using the individual metal solution (Cu, Zn, Cr, Co, and Ni) demonstrated an increase in antimicrobial activity which correlated with an increased metal ion solution concentration (*P* < 0.001) (Figure 7). Thus, ZOI assays of metals demonstrated *S. aureus* (ZOI: 25.4 mm) as the most sensitive CM pathogens followed by *Bacillus* (ZOI: 23.4 mm), *E. coli* (ZOI: 20.6 mm), *Enterobacter* (ZOI: 18.9 mm), *Klebsiella* (ZOI: 17.8 mm), and *Shigella* (ZOI: 15.4 mm) (Figure 7A). The minimal inhibitory concentration (MIC) of the metal ions demonstrated a varying degree of response against all the tested CM pathogens, and these bacteria tolerated a wide range of metal concentration (3.4–38.1 μg/mL) (Supplementary Material). We compared the highest MIC values of each metal, and found that the highest MIC values decrease in the following order: Zn (38.1 μg/mL, *S. aureus*), Cu (33.2 μg/mL, *S. aureus*), Ni (28.2 μg/mL, *E. coli*), Cr (17.2 μg/mL, *Enterobacter* species), and Co (15.3 μg/mL, *Bacillus* spp.) (Figure 7B and Supplementary Material). For the MIC of specific bacteria, the most effective metals were found to be Cr against *Shigella* (3.4 μg/mL) and *Klebsiella* (5.8 μg/mL) species, Ni against *Shigella* (3.5 μg/mL) species, Co against *Shigella* (5 μg/mL) and *Klebsiella* (7.4 μg/mL) species, and Cu and Zn against *Shigella* (7.5 μg/mL, both) species. In contrast, Zn (38.1 μg/mL) and Cu (33.2 μg/mL) were the least toxic metals against *S. aureus* (Figure 7B and Supplementary Material). A similar pattern was demonstrated for the minimal bactericidal concentration (MBC) with the greatest bactericidal activity for Cr against *S. aureus* (11.3 μg/mL) followed by Co against *E. coli* (14.3 μg/mL), Ni against *S. aureus* (23.1 μg/mL), Zn against *E. coli* (24.2 μg/mL), and Cu against *Shigella* (25.1 μg/mL) species. However, Cu produced equable antimicrobial efficacy as Zn, Cr, Co and Ni against *Enterobacter* species (≤25.5 μg/mL) (Supplementary Table S2).

**FIGURE 8:**
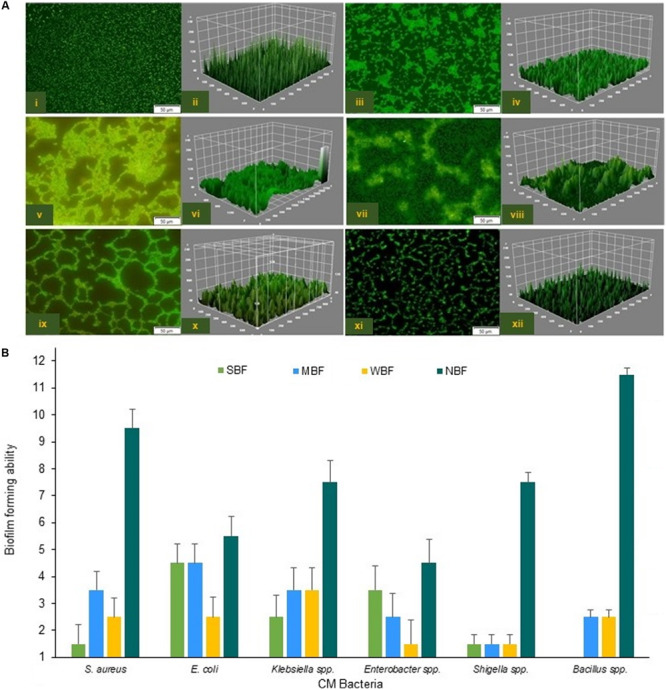
Biofilm formation (BF) ability of the six CM causing pathogens. **(A)** Confocal fluorescence images (2D and 3D) of *S. aureus* (i,ii), *E. coli* (iii,iv), *Klebsiella* spp. (v,vi), *Enterobacter* spp. (vii,viii), *Bacillus* spp. (ix,x) and *Shigella* spp. (xi,xii). Scale bars are indicated in μm. **(B)** Capability of the biofilm formation by six CM causing bacteria.

To assess the BF ability of CM pathogens in *in vitro* conditions, we randomly selected 80 isolates (*S. aureus*, 15; *E. coli*, 15; *Klebsiella*, 15; *Bacillus*, 15; *Enterobacter*, 10 and *Shigella*, 10) for a BF assay. In this study, 76.2% (61/80) the bacterial species were biofilm-formers with significance differences (*P* = 0.028) in their BF categories, which were designated as strong biofilm forming (SBF, 28.7%), moderate biofilm forming (MBF, 25.2%), weak biofilm forming (WBF, 22.2%), and non-biofilm forming (NBF, 23.7%) (Figure 8). Microscopic observation followed by 3D image analysis revealed that the intensity of green fluorescence remained higher, indicating that a large number of cells were viable and attached to the surface (Figure 8A). While investigated individually, *E. coli* (66.7%) remained as the highest biofilm producing CM pathogen followed by *Enterobacter* (60.0%), *Klebsiella* (46.7%), *S. aureus* (40.0%), *Shigella* (30.0%), and *Bacillus* (26.7%) species. Our current findings reveal that Gram-negative CM pathogens (*Enterobacter*, 60.0%; *E. coli*, 40.0%; *Shigella*, 33.3%; *Klebsiella*, 28.6%) have a higher biofilm producing ability than Gram-positive bacteria (*S. aureus*, 16.7%) (Figures 8A,B). On the contrary, the majority of the *Bacillus* (73.3%), *Shigella* (70.0%), and *S. aureus* (60.0%) isolates remained as non-biofilm formers (NBF) (Figure 8B). Therefore, our current findings of *in vitro* resistance analysis (antibiotics and metals resistance and biofilm assays) corroborate the resistome found in metagenome sequencing.

### Microbiome Functional Analysis

**FIGURE 9:**
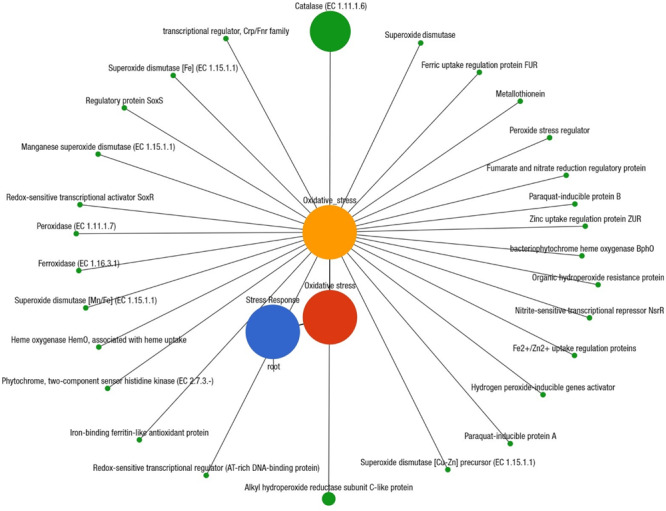
Projection of the clinical mastitis (CM) milk metagenome onto KEGG pathways. The whole metagenome sequencing (WMS) reveals significant differences (Kruskal–Wallis test, *P* = 0.001) in functional microbial pathways. A total of 28 genes associated with oxidative stress were found in CM microbiomes. Black lines with green circles delineate the distribution of the stress related genes according to their class across the CM metagenome. The diameter of the circles indicates the relative abundance of the respective genes. More details about these genes can be found in the text and Supplementary Material.

We also investigated the possible links between chemotaxis and pathogenicity through the identification of putative genes or proteins associated with both flagellar motility and bacterial chemotaxis. The KEGG pathway analysis identified 48 protein families associated with flagellar motility in prokaryotes, and among them, flagellar hook-length control protein, FliK (27.1%); flagellar biosynthesis proteins, FlhA, FliL, FliP, FlhF, FlgN, FliS, FlhB, FliO, FliQ (∼16.0%); flagellar M-ring protein, FliF (5.6%); and flagellar regulatory protein, FleQ (5.3%) were predominantly associated with cell motility (Supplementary Material). Twenty-six functional genes encoding different proteins were found to be associated with bacterial chemotaxis (Supplementary Figure S6 and Supplementary Material), of them, methyl-accepting chemotaxis protein, mcp (44.2%); chemotaxis family proteins of bacterial two component system, CheV, CheA, CheB, CheBR, CheY (∼15.0%); aerotaxis receptor, Aer (7.5%); MotB (5.2%), and MotA (3.1%) were the most abundant among these CM microbiotas (Supplementary Material). To explore the role of regulation and cell signaling mechanisms in mammary gland pathogenesis, using the SEED subsystem module of MR analysis, we found two-component regulatory systems BarA-UvrYBarA-UvrY (*sir*A) as the most abundant virulence regulatory gene (84.1%) in CM microbiomes (Supplementary Material). Another regulatory and cell signaling gene, endoplasmic reticulum chaperon *grp*78 (BiP), was also found as the single most abundant (93.8%) gene in the proteolytic pathways of the CM associated bacterial strains (Supplementary Figure S7 and Supplementary Material). A deeper look at microbial genes associated with phages-prophages, transposable elements, and plasmids revealed that pathogenicity islands related proteins such as methionine-ABC transporter substrate-binding protein (33.8%), GMP synthase (27.7%), tmRNA-binding protein; SmpB (16.0%), heat shock protein 60; GroEL (16.0%), and SSU ribosomal protein; S18p (6.1%) were predominantly abundant among the CM pathogens (Supplementary Material). We also found significant associations between the number of reads assigned to genes coding for AMR and biofilm-formation-and-quorum-sensing (Pearson correlation, *P* = 0.0001), and in the relative abundance of genes coding for biofilm-formation and flagellar activities (Pearson correlation, *P* = 0.004). The SEED module analysis also enabled us to identify 28 different protein functions associated with oxidative stress responses that were mostly represented by catalase related proteins (26.7%), Cu-Zn-Fe-Mn mediated superoxide dismutase (12.7%), H_2_O_2_-inducible genes activator (7.8%), and paraquat-inducible protein B (7.3%) (Figure 9 and Supplementary Material).

## Discussion

Previously, we reported that the bovine CM milk microbiome harbors genes that may promote microbial pathogenicity, including AMR compared to the healthy state (Hoque et al., 2019). In this study, we employed a combination of both *in silico* (WMS) and *in vitro* (culture-based) approaches to elucidate the resistance potentials in CM associated microbiomes. Currently, it is feasible to use metagenomic sequencing as a diagnostic tool for the detection of microbiome and its associated resistome in environmental samples (Pérez-Cobas et al., 2013; Schaik, 2015; Lanza et al., 2018). Shotgun metagenomics (WMS) investigations are progressively being used to analyze the ensemble of genes that may encode antibiotic resistance in various microbial ecosystems, which are defined as the resistome (Forsberg et al., 2012; Schaik, 2015; Su et al., 2017; Lanza et al., 2018; Zaheer et al., 2018). The increasing prevalence of AMR in bacteria in bovine CM milk is one of the most important challenges that public health faces. It has been proposed that livestock production systems may contribute to an increased prevalence of antimicrobial resistant bacteria, and/or associated genes in the environment and therefore pose a risk to human health. Globally, more than 57 million kilograms of antibiotics are used annually in food animal production (Van Boeckel et al., 2015), which may select for antibiotic-resistant bacteria that persist throughout the meat and milk production chain. Investigation of the microbiome, and/or associated resistome of dairy animals especially in milk from cows with mastitis, and their environment may provide valuable data and models to estimate the public health risk of antibiotic-resistant human infections associated with antibiotic use in dairy animals (Zaheer et al., 2019).

Our present findings are sufficiently enriched in taxonomic resolution and predicted protein functions, and corroborates the findings of several previous studies (Oikonomou et al., 2014; Falentin et al., 2016; Patel et al., 2017; Hoque et al., 2019). The occurrence of bovine mastitis could be affected by cattle breeds (Cremonesi et al., 2018; Curone et al., 2018; Gonzalez-Recio et al., 2018), and the diversity of CM-causing pathogens is associated with a broad range of host-defense mechanisms as part of its immunological arsenal (Thompson-Crispi et al., 2014; Li et al., 2019). We found significant differences in taxonomic diversity and abundances among the CM microbiomes of four dairy breeds. The XHF cows suffering from CM had higher microbial diversity at strain-level, and a significant proportion of the microbiota found to be shared with that of the other three breeds (LZ, SW, and RCC). Consistent with the results of earlier studies (Cremonesi et al., 2018; Curone et al., 2018; Gonzalez-Recio et al., 2018; Li et al., 2019), the taxonomic profile of the CM microbiomes found in four breeds of cows were dominated by phyla Proteobacteria, Bacteroidetes, Firmicutes, Actinobacteria, and Fusobacteria. This breed specific variation in taxonomic richness and diversity of the microbiome, especially in XHF and LZ cows, could be associated with their increased disease resistance or immune response (Cremonesi et al., 2018; Curone et al., 2018; Gonzalez-Recio et al., 2018), and rumen microbial features (e.g., taxa, diversity indices, functional categories, and genes) (Li et al., 2019). However, this study demonstrated that the resistome is not significantly correlated with different dairy breeds suggesting that resistance potentials of CM microbiome might be common among dairy breeds. In our study, lack of congruence between resistome and microbial communities in four dairy breeds suggested that other factors, in addition to antimicrobial use, might be associated with changes in resistome, and microbiome composition. It may also reflect the possibility that a small fraction of the total bacterial population was resistant to antibiotics (MacLean and Vogwill, 2015; Rovira et al., 2019), and that horizontal gene transfer disrupted the link between microbiome and resistome composition (Johnson et al., 2015). However, further investigations are necessary to evaluate the real effect of breed specific bacteria on cow mammary gland diseases. Recent understandings regarding evolutionary relationships of major CM causing bacteria are primarily based on 16S rRNA gene phylogenetic identification along with a few individual gene or protein sequences (Naushad et al., 2016), which often produces conflicting phylogenies. This study explored the possibility that the prevalence of CM milk pathogens could vary according to geographical locations, and farming (semi-intensive to intensive grazing system in SER, semi-intensive to free-range grazing systems in CR) systems (Reyes-Jara et al., 2016). These differences may imply that the etiology of bovine CM in Bangladesh could be related to the breed/host genetic factors (Cremonesi et al., 2018; Curone et al., 2018; Gonzalez-Recio et al., 2018; Li et al., 2019), types of feeding, and farm locations and types (Reyes-Jara et al., 2016), types of antibiotics and/or metals used for treatment resistomes, and other predisposing factors, as described in other countries (Preethirani et al., 2015; Reyes-Jara et al., 2016; Cheng et al., 2019).

Data presented here, coupled with the data reported in our earlier study (Hoque et al., 2019) provide important insights into the resistance potentials in CM microbiomes. Our results are concordant with MDR bacteria reported elsewhere from the milk of clinically infected cows (Curone et al., 2018; Tomazi et al., 2018; Cheng et al., 2019), buffalo cows (Preethirani et al., 2015), and humans (Penders et al., 2013; Patel et al., 2017; Baron et al., 2018). Our findings linked multidrug resistance to efflux pumps (MREP), *Cme*ABC operon, *mdt*ABCD cluster, *Bla*R1 family, methicillin resistance in *Staphylococcus* (MRS), resistance to fluoroquinolones (RFL), and multiple metals resistance to CZCR and AR as the predominantly abundant antibiotics and toxic compounds resistance (RATC) functional groups in CM microbiomes, suggesting that bovine CM milk microbiome constitutes a good reservoir for AMR (Kumar and Varela, 2012; Penders et al., 2013; Baron et al., 2018; Hoque et al., 2019). It has been reported that efflux pumps regulated by two-component systems in several pathogens, including *A. baumannii* and *K. pneumonia*, provide multidrug resistance, which may limit the treatment options against bacterial infections of the mammary glands (Li and Nikaido, 2009; Tiwari et al., 2017). Relative over-expression of efflux pumps enhances resistance to antimicrobials by reducing the accumulation of antibiotics inside the bacterial cells, providing sufficient time for the bacteria to adapt to the antibiotics (slow phase antibiotic efflux), and through mutations or alteration of antibiotic targets (Yao et al., 2016; Tiwari et al., 2017). The *Cme*ABC operon is highly potent against multiple antibiotics, promotes the emergence of ARGs, and confers exceedingly high-level resistance to fluoroquinolones (Singh et al., 2017). Therefore, multidrug resistance to efflux pumps and multiple heavy metals resistance represented ubiquitous resistance mechanisms among CM microbiomes, which might be associated with unethical overuse of antibiotics in dairy animals (Preethirani et al., 2015; Curone et al., 2018; Tomazi et al., 2018; Cheng et al., 2019; Zaheer et al., 2019), and extensive application of toxic chemicals and metals in agricultural use (Reyes-Jara et al., 2016; Vaidya et al., 2017), or might have a function in the gut microbiome that is still unknown (Penders et al., 2013; Hu et al., 2014b; Ciesinski et al., 2018). The RATC genes detected in this study are of particular interest because there is concern that the use of this class of antibiotics or metals in veterinary medicine, particularly for food animals, may contribute to the development of resistance to this class of antimicrobial options in humans (Penders et al., 2013; Hu et al., 2014b).

The *in vitro* antibiogram assays revealed that a high proportion of multidrug resistant (MDR) bacteria was frequently observed, where tetracyclines (tetracycline and doxycycline), quinolones (nalidixic acid), penicillins (ampicillin), and phenols (chloramphenicol) were the most common resistant antibiotics against the six selected CM pathogens. This finding of high MDR patterns for CM pathogens is in line with many previous studies on bovine mastitis (Preethirani et al., 2015; Cheng et al., 2019). The AMR profile of bovine CM pathogens for different antimicrobials could vary according to the type and origin of bacteria (Preethirani et al., 2015; Van Boeckel et al., 2015; Cheng et al., 2019) and host-population such as bovine (Tomazi et al., 2018; Cheng et al., 2019) and bubaline (Preethirani et al., 2015) cows. Consistent with bacterial needs, heavy metals can be transformed (e.g., oxidized, reduced, methylated, or complexed) and used as a source of energy, terminal electron acceptors, and/or enzyme structural elements (Drewniak et al., 2016). The highest abundance of CZCR genes among CM pathogens is mainly due to the presence of Co, Zn, and Cd detoxification systems (Drewniak et al., 2016). Although understanding of the uncontrolled spread of ARGs in bovine mastitis pathogens (Cheng et al., 2019) is growing, information on toxic compounds or heavy metal resistance remains unavailable. In this study, heavy metals (Cr, Co, Ni, and Cu) tested for antibacterial sensitivity showed good efficacy, but knowledge on their mode of action is limited. Thus, with the increase of MDR bacteria in CM, there is an imperative need for new biocidal and antimicrobial formulations. The MIC and MBC tested metals revealed effective antimicrobial efficacies against a wide range of AMR pathogens (Reyes-Jara et al., 2016; Vaidya et al., 2017; Ciesinski et al., 2018). We found that Cr and Co compounds had the highest antimicrobial efficacy (MIC) against all of the tested bacteria as supported by several previous studies (Vaidya et al., 2017; Murcia et al., 2018). This finding is likely related to the better lipophilicity of Cr and Co, resulting in increased antimicrobial activities or chelating effects (Murcia et al., 2018). However, both Cr and Co, should be used cautiously either in medication or in feed supplementation after calculating appropriate concentrations, to avoid cytotoxicity. These *in vitro* resistance patterns of CM bacteria corroborate the resistome profile of the WMS, since multidrug resistance to efflux pumps, resistance to fluoroquinolones, beta lactamases, and methicillin were the predominant antibiotics resistance functional groups, whereas cobalt, zinc, cadmium, copper, arsenic, and chromium resistance were the predominating toxic metals resistant genes in CM causing microbiomes. Molecular methods complemented with culture-based diagnostics have been historically implemented to document AMR in bacteria, however, the advent of shotgun metagenomics (WMS) has revolutionized the method of addressing relevant problems like diagnosis and surveillance of infectious diseases, and the issue of AMR (De, 2019). Biofilm formation is an important virulence factor that may result in recurrent or persistent udder infections (Melchior et al., 2006), and treatment failure through increased resistance to antibiotics and protection against host defenses (Schönborn et al., 2017). The relative overexpression of genes encoding *lsr*ACDBFGE operon, biofilm adhesion biosynthesis, protein *Yjg*K cluster, and QS: autoinducer-2 synthesis in CM microbiomes is in accordance with several earlier reports (Gomes et al., 2016; Schönborn et al., 2017; Hoque et al., 2019). In this study, the relative abundance of the predicted proteins for BF and QS varied significantly among the six selected bacterial taxa. Bacterial BF and QS abilities can be the strain specific or genetically linked traits, representing a selective advantage in pathogenesis of bovine CM (Schönborn et al., 2017). Biofilms can enhance proliferation of reactive oxygen and nitrogen species that can survive antibiotic treatment leading to the transfer of ARGs (Gomes et al., 2016). In this study, overall, 76.2% of the isolates of the six selected CM pathogens were found as biofilm producers, and their ability to produce biofilm varied significantly (Gomes et al., 2016; Schönborn et al., 2017). A large number of food spoilage and/or pathogenic bacteria, including *Enterococcus faecalis*, *Enterobacter* spp., *Pseudomonas* spp., *Klebsiella* spp., *S. aureus*, *E. coli*, *B. cereus*, and others, have already been reported to be associated with biofilm-formation (BF) from diary niches (Melchior et al., 2006; Gomes et al., 2016; Schönborn et al., 2017; Singh et al., 2017; Vaidya et al., 2017) which supports our current findings.

Bacterial chemotaxis mediated by flagellar activities (Duan et al., 2013) and the flagella mediated virulence factors are found in many pathogenic species of bovine CM microbiomes, making them a potential target for new antibacterial therapeutics (Duan et al., 2013). The intra- and interspecies cell-to-cell communication in bovine CM microbiomes were associated with 26 different genes, which might have vital roles in the early phase of mastitis for attachment to, or entry into the udder tissues and virulence regulation (Zatakia et al., 2018), and bacterial colonization in mammary tissues like other suitable sites (Matilla and Krell, 2017). The *cheA-cheY* two-component system mediated bacterial chemotaxis also facilitates the initial contact of bacteria with mammary gland epithelial cells, and contribute to effective invasion (Dons et al., 2004). The two-component signal transduction system *BarA-UvrY* regulates metabolism, motility, BF, stress resistance, virulence, and QS in CM pathogens by activating the transcription of genes for regulatory small RNAs (Zere et al., 2015). The up-regulation of genes coding for proteolytic activity, *grp78* during host-pathogen interactions in CM, is associated with endoplasmic reticulum (ER) stress which further triggers proteolytic activities to initiate the mechanism of pathogenesis and cell death (Hirai et al., 2018). Catalase activity is a marker of bovine mastitis, which plays a central role in milk redox control and markedly increases during the pathophysiology of bovine CM (Andrei et al., 2016). Our present findings are in line with previous reports (Andrei et al., 2016; Darbaz et al., 2019) that an elevated oxidative stress mediated by catalase activity might have originated either from the mammary gland and/or bacterial cells. During the pathogenesis of bovine mammary gland, bacteria are not rapidly killed by the phagocytic activity of bovine macrophages; rather, they survive within macrophages during prolonged infection due to secretion of catalase and superoxide dismutase, which by degrading H_2_O_2_, inhibit the ROS mediated killing mechanism of the host (Andrei et al., 2016; Darbaz et al., 2019). The majority of published studies on bovine mastitis describe 16S rRNA gene-based community structure evaluations, whereas published reports on shotgun deep sequencing metagenomics of CM microbiome studies remain scarce. We completed an in-depth report simultaneously describing microbiome diversity along with previously unreported opportunistic strains, and their associated resistome in bovine mastitis.

## Conclusion

Bovine CM milk is a potential reservoir of diverse groups of microbes harboring a diverse resistome and other virulence factors. Our findings reveal that genes coding for multidrug and multiple metal resistance are ubiquitously present in the CM microbiome, and protect bacteria from the antibacterial effects of antimicrobials, extruding them out of cells. The efflux pumps mediated by multidrug resistance and multiple metals (e.g., cobalt, zinc, cadmium, arsenic, chromium) resistance were the predominating genomic functional groups to be considered as the potential key factors for the persistence of bovine CM, and the failure of conventional therapies against CM-related pathogens. Additionally, BF, QS, bacterial flagellar movement and chemotaxis, regulation and cell signaling, and oxidative stress associated genes can be a great benefit to bacteria against the host’s immune system. Microbial resistance and genomic functional potentials represent important mechanisms for antibiotic resistance, leading to treatment failure and persistence of the disease. An accurate and timely identification of CM-associated pathogens and analyses of their resistance potentials are necessary for the selection of proper therapeutics (antibiotics and/or metals), and the development of effective, safe, and economical treatment regimens for bovine mastitis and sustainable dairying. Although the baseline data presented here are promising, further studies are recommended using a larger sample size, and with the inclusion of gut microbiome sampling in addition to the milk samples to elucidate the specific resistance potential of CM pathogens as well as for direct testing of microbiome and resistome transfer across this axis.

## Data Availability Statement

The sequence data reported in this article have been deposited in the NCBI database (BioProject PRJNA529353 for metagenome sequences, NCBI accession numbers: MN 620423–MN 620430 for 16S rRNA gene sequences).

## Ethics Statement

The protocol for milk sample collection from lactating dairy cows was approved by the Animal Experimentation Ethical Review Committee (AEERC), Faculty of Biological Sciences, University of Dhaka.

## Author Contributions

MNH, MS, AI, and MAH conceived and designed the overall study. MNH surveyed and collected all the field samples. MNH, RC, KG, OS, and OI carried out laboratory work including DNA extractions and sequencing, microbiological (cultural, biochemical) examinations, antimicrobial (antibiotics, metals) sensitivity tests, and biofilm assays. MAH and KC contributed chemicals and reagents. MNH, RA, and AI conceived, designed and executed the bioinformatics analysis. MNH interpreted the results and drafted the manuscript. MS, AS, KC, and MAH contributed intellectually to the interpretation and presentation of the results. Finally, all authors have approved the manuscript for submission.

## Conflict of Interest

The authors declare that the research was conducted in the absence of any commercial or financial relationships that could be construed as a potential conflict of interest.

## References

[B1] Abebe R. Hatiya H. Abera M. Megersa B. Asmare K. (2016). Bovine mastitis: prevalence, risk factors and isolation of *Staphylococcus aureus* in dairy herds at Hawassa milk shed, South Ethiopia. *BMC Vet. Res.* 12:270. 10.1186/s12917-016-0905-3 27912754 PMC5135792

[B2] Andrei S. Matei S. Rugină D. Bogdan L. Ştefănuţ C. (2016). Interrelationships between the content of oxidative markers, antioxidative status, and somatic cell count in cow’s milk. *Czech J. Animal Sci.* 61 407–413. 10.17221/70/2015-CJAS

[B3] Baron S. Diene S. Rolain J. M. (2018). Human microbiomes and antibiotic resistance. *Hum. Microb J.* 10 43–52. 10.1016/j.humic.2018.08.005

[B4] Beck J. Holloway J. D. Schwanghart W. (2013). Under sampling and the measurement of beta diversity. *Methods Ecol. Evol.* 4 370–382. 10.1111/2041-210x.12023 11767643

[B5] Cheng J. Qu W. Barkema H. W. Nobrega D. B. Gao J. Liu G. (2019). Antimicrobial resistance profiles of 5 common bovine mastitis pathogens in large Chinese dairy herds. *J. Dairy Sci.* 102 1–11. 10.3168/jds.2018-15135 30639013

[B6] Ciesinski L. Guenther S. Pieper R. Kalisch M. Bednorz C. Wieler L. H. (2018). High dietary zinc feeding promotes persistence of multi-resistant *E. coli* in the swine gut. *PLoS One* 13:e0191660. 10.1371/journal.pone.0191660 29373597 PMC5786291

[B7] CLSI (2017). *Performance standards for antimicrobial susceptibility testing; CLSI document M100–S27.* Wayne, PA: Clinical Laboratory Standards Institute (CLSI).

[B8] Cremonesi P. Ceccarani C. Curone G. Severgnini M. Pollera C. Bronzo V. (2018). Milk microbiome diversity and bacterial group prevalence in a comparison between healthy Holstein Friesian and Rendena cows. *PLoS One* 13:e0205054. 10.1371/journal.pone.0205054 30356246 PMC6200206

[B9] Curone G. Filipe J. Cremonesi P. Trevisi E. Amadori M. Pollera C. (2018). What we have lost: mastitis resistance in Holstein Friesians and in a local cattle breed. *Res. Vet. Sci.* 116 88–98. 10.1016/j.rvsc.2017.11.020 29223308

[B10] Darbaz I. Salar S. Sayiner S. Bastan I. Ergene O. Bastan A. (2019). Evaluation of milk glutathione peroxidase and superoxide dismutase levels in subclinical mastitis in Damascus goats. *Turk. J. Vet. Anim. Sci.* 43 259–263. 10.3906/vet-1810-60 31411186

[B11] D’Costa V. M. King C. E. Kalan L. Morar M. Sung W. W. Schwarz C. (2011). Antibiotic resistance is ancient. *Nature* 477 457–461. 10.1038/nature10388 21881561

[B12] De R. (2019). Metagenomics: aid to combat antimicrobial resistance in diarrhea. *Gut Pathog.* 11 1–9. 10.1186/s13099-019-0331-8 31636714 PMC6791012

[B13] Dons L. Eriksson E. Jin Y. Rottenberg M. E. Kristensson K. Larsen C. N. (2004). Role of flagellin and the two-component CheA/CheY system of *Listeria monocytogenes* in host cell invasion and virulence. *Infect. Immun.* 72 3237–3244. 10.1128/IAI.72.6.3237-3244.2004 15155625 PMC415653

[B14] Drewniak L. Krawczyk P. S. Mielnicki S. Adamska D. Sobczak A. Lipinski L. (2016). Physiological and metagenomic analyses of microbial mats involved in self-purification of mine waters contaminated with heavy metals. *Front. Microbiol.* 7:1252. 10.3389/fmicb.2016.01252 27559332 PMC4978725

[B15] Duan Q. Zhou M. Zhu L. Zhu G. (2013). Flagella and bacterial pathogenicity. *J. Basic Microbiol.* 53 1–8. 10.1002/jobm.201100335 22359233

[B16] Falentin H. Rault L. Nicolas A. Bouchard D. S. Lassalas J. Lamberton P. (2016). Bovine teat microbiome analysis revealed reduced alpha diversity and significant changes in taxonomic profiles in quarters with a history of mastitis. *Front. Microbiol.* 7:480. 10.3389/fmicb.2016.00480 27242672 PMC4876361

[B17] Forsberg K. J. Reyes A. Wang B. Selleck E. M. Sommer M. O. Dantas G. (2012). The shared antibiotic resistome of soil bacteria and human pathogens. *Science* 337 1107–1111. 10.1126/science.1220761 22936781 PMC4070369

[B18] Francis O. E. Bendall M. Manimaran S. Hong C. Clement N. L. Castro-Nallar E. (2013). Pathoscope: species identification and strain attribution with unassembled sequencing data. *Genome Res.* 23 1721–1729. 10.1101/gr.150151.112 23843222 PMC3787268

[B19] Gao J. Barkema H. W. Zhang L. Liu G. Deng Z. Cai L. (2017). Incidence of clinical mastitis and distribution of pathogens on large Chinese dairy farms. *J. Dairy Sci.* 100 4797–4806. 10.3168/jds.2016-12334 28434736

[B20] Glass E. M. Wilkening J. Wilke A. Antonopoulos D. Meyer F. (2010). Using the metagenomics RAST server (MG-RAST) for analyzing shotgun metagenomes. *Cold Spring Harb Protoc.* 2010:5368. 10.1101/pdb.prot5368 20150127

[B21] Gomes F. Saavedra M. J. Henriques M. (2016). Bovine mastitis disease/pathogenicity: evidence of the potential role of microbial biofilms. *Patho. Dis.* 74 1–7. 10.1093/femspd/ftw006 26772653

[B22] Gonzalez-Recio O. Zubiria I. García-Rodríguez A. Hurtado A. Atxaerandio R. (2018). Signs of host genetic regulation in the microbiome composition in 2 dairy breeds: holstein and Brown Swiss. *J. Dairy Sci.* 101 2285–2292. 10.3168/jds.2017-13179 29274973

[B23] Head S. R. Komori H. K. LaMere S. A. Whisenant T. Van Nieuwerburgh F. Salomon D. R. (2014). Library construction for next-generation sequencing: overviews and challenges. *Biotechniques* 56 61–77. 10.2144/000114133 24502796 PMC4351865

[B24] Hirai K. E. de Sousa J. R. Silva L. M. Junior L. B. D. Furlaneto I. P. Carneiro F. R. O. (2018). Endoplasmic reticulum stress markers and their possible implications in leprosy’s pathogenesis. *Dis. Markers.* 2018:7067961. 10.1155/2018/7067961 30647798 PMC6311872

[B25] Hong C. Manimaran S. Shen Y. Perez-Rogers J. F. Byrd A. L. Castro-Nallar E. (2014). PathoScope 2.0: a complete computational framework for strain identification in environmental or clinical sequencing samples. *Microbiome* 2:33. 10.1186/2049-2618-2-33 25225611 PMC4164323

[B26] Hoque M. N. Das Z. C. Rahman A. N. M. A. Haider M. G. Islam M. A. (2018). Molecular characterization of *Staphylococcus aureus* strains in bovine mastitis milk in Bangladesh. *Intl. J. Vet. Sci. Med.* 6 53–60. 10.1016/j.ijvsm.2018.03.008 30255079 PMC6147393

[B27] Hoque M. N. Das Z. C. Talukder A. K. Alam M. S. Rahman A. N. M. A. (2015). Different screening tests and milk somatic cell count for the prevalence of subclinical bovine mastitis in Bangladesh. *Trop. Anim. Health Prod.* 47 79–86. 10.1007/s11250-014-0688-0 25326717

[B28] Hoque M. N. Istiaq A. Clement R. A. Sultana M. Siddiki A. M. A. M. Z. Crandall K. A. (2019). Metagenomic deep sequencing reveals association of microbiome signature with functional biases in bovine mastitis. *Sci. Rep.* 9:13536. 10.1038/s41598-019-49468-4 31537825 PMC6753130

[B29] Hu Y. Yan C. Hsu C. H. Chen Q. R. Niu K. Komatsoulis G. A. (2014a). OmicCircos: a simple-to-use R package for the circular visualization of multidimensional omics data. *Cancer Inform.* 13 3–20. 10.4137/CIN.S13495 24526832 PMC3921174

[B30] Hu Y. Yang X. Lu N. Zhu B. (2014b). The abundance of antibiotic resistance genes in human guts has correlation to the consumption of antibiotics in animal. *Gut Microb.* 5 245–249. 10.4161/gmic.27916 24637798 PMC4063852

[B31] Johnson T. A. Stedtfeld R. D. Wang Q. Cole J. R. Hashsham S. A. Looft T. (2015). Clusters of antibiotic resistance genes enriched together stay together in swine agriculture. *mBio* 7:e02214-15. 10.1128/mBio.02214-15 27073098 PMC4959523

[B32] Kanehisa M. Sato Y. Furumichi M. Morishima K. Tanabe M. (2019). New approach for understanding genome variations in KEGG. *Nucleic Acids Res.* 47 D590–D595. 10.1093/nar/gky962 30321428 PMC6324070

[B33] Koh H. (2018). An adaptive microbiome α-diversity-based association analysis method. *Sci. Rep.* 8:18026. 10.1038/s41598-018-36355-7 30575793 PMC6303306

[B34] Krömker V. Leimbach S. (2017). Mastitis treatment-Reduction in antibiotic usage in dairy cows. *Reprod. Domestic Anim.* 52 21–29. 10.1111/rda.13032 28815847

[B35] Kumar S. Stecher G. Tamura K. (2016). MEGA7: molecular evolutionary genetics analysis version 7.0 for bigger datasets. *Mol. Biol. Evol.* 33 1870–1874. 10.1093/molbev/msw054 27004904 PMC8210823

[B36] Kumar S. Varela M. F. (2012). Biochemistry of bacterial multidrug efflux pumps. *Intl. J. Mol. Sci.* 13 4484–4495. 10.3390/ijms13044484 22605991 PMC3344227

[B37] Langmead B. Salzberg S. L. (2012). Fast gapped-read alignment with Bowtie 2. *Nat. Methods* 9 357–359. 10.1038/nmeth.1923 22388286 PMC3322381

[B38] Lanza V. F. Baquero F. Martínez J. L. Ramos-Ruiz R. Gonzalez-Zorn B. Andremont A. (2018). In-depth resistome analysis by targeted metagenomics. *Microbiome* 6:11. 10.1186/s40168-017-0387-y 29335005 PMC5769438

[B39] Larkin M. A. Blackshields G. Brown N. P. Chenna R. McGettigan P. A. McWilliam H. (2007). Clustal W and Clustal X version 2.0. *Bioinformatics* 23 2947–2948. 10.1093/bioinformatics/btm404 17846036

[B40] Letunic I. Bork P. (2011). Interactive Tree Of Life v2: online annotation and display of phylogenetic trees made easy. *Nucleic Acids Res.* 39 W475–W478. 10.1093/nar/gkw290 21470960 PMC3125724

[B41] Li F. Li C. Chen Y. Liu J. Zhang C. Irving B. (2019). Host genetics influence the rumen microbiota and heritable rumen microbial features associate with feed efficiency in cattle. *Microbiome* 7:92. 10.1186/s40168-019-0699-1 31196178 PMC6567441

[B42] Li X. Z. Nikaido H. (2009). Efflux-mediated drug resistance in bacteria: an update. *Drugs* 69 1555–1623. 10.2165/11317030-000000000-00000 19678712 PMC2847397

[B43] MacLean R. C. Vogwill T. (2015). Limits to compensatory adaptation and the persistence of antibiotic resistance in pathogenic bacteria. *Evol. Med. Public Health* 2015 4–12. 10.1093/emph/eou032 25535278 PMC4323496

[B44] Masomian M. Rahman R. N. Z. R. A. Salleh A. B. Basri M. (2016). Analysis of comparative sequence and genomic data to verify phylogenetic relationship and explore a new subfamily of bacterial lipases. *PLoS One* 11:e0149851. 10.1371/journal.pone.0149851 26934700 PMC4774917

[B45] Matilla M. A. Krell T. (2017). The effect of bacterial chemotaxis on host infection and pathogenicity. *FEMS Microbiol. Rev.* 42:fux052. 10.1093/femsre/fux052 29069367

[B46] McMurdie P. J. Susan H. (2013). Phyloseq: an R package for reproducible interactive analysis and graphics of microbiome census data. *PLoS One* 8:e61217. 10.1371/journal.pone.0061217 23630581 PMC3632530

[B47] Melchior M. B. Vaarkamp H. Fink-Gremmels J. (2006). Biofilms: a role in recurrent mastitis infections? *Vet. J.* 171 398–407. 10.1016/j.tvjl.2005.01.006 16624706

[B48] Murcia R. Leal S. M. Roa M. V. Nagles E. Muñoz-Castro A. Hurtado J. J. (2018). Development of antibacterial and antifungal triazole chromium (III) and cobalt (II) complexes: synthesis and biological activity evaluations. *Molecules* 23:2013. 10.3390/molecules23082013 30104466 PMC6222626

[B49] Naushad S. Barkema H. W. Luby C. Condas L. A. Nobrega D. B. Carson D. A. (2016). Comprehensive phylogenetic analysis of bovine non-aureus staphylococci species based on whole-genome sequencing. *Front. Microbiol.* 7:1990. 10.3389/fmicb.2016.01990 28066335 PMC5168469

[B50] Oikonomou G. Bicalho M. L. Meira E. Rossi R. E. Foditsch C. Machado V. S. (2014). Microbiota of cow’s milk; distinguishing healthy, subclinically and clinically diseased quarters. *PLoS One* 9:e85904. 10.1371/journal.pone.0085904 24465777 PMC3896433

[B51] Oniciuc E. Likotrafiti E. Alvarez-Molina A. Prieto M. Santos J. A. Alvarez-Ordóñez A. (2018). The present and future of Whole Genome Sequencing (WGS) and Whole Metagenome Sequencing (WMS) for surveillance of antimicrobial resistant microorganisms and antimicrobial resistance genes across the food chain. *Genes* 9:268. 10.3390/genes9070315 29789467 PMC5977208

[B52] Patel S. H. Vaidya Y. H. Patel R. J. Pandit R. J. Joshi C. G. Kunjadiya A. P. (2017). Culture independent assessment of human milk microbial community in lactational mastitis. *Sci. Rep.* 7:7804. 10.1038/s41598-017-08451-7 28798374 PMC5552812

[B53] Pattengale N. D. Alipour M. Bininda-Emonds O. R. Moret B. M. Stamatakis A. (2010). How many bootstrap replicates are necessary? *J. Comput. Biol.* 17 337–354. 10.1089/cmb.2009.0179 20377449

[B54] Penders J. Stobberingh E. Savelkoul P. Wolffs P. (2013). The human microbiome as a reservoir of antimicrobial resistance. *Front. Microbiol.* 4:87. 10.3389/fmicb.2013.00087 23616784 PMC3627978

[B55] Pérez-Cobas A. E. Artacho A. Knecht H. Ferrús M. L. Friedrichs A. Ott S. J. (2013). Differential effects of antibiotic therapy on the structure and function of human gut microbiota. *PLoS One* 8:e80201. 10.1371/journal.pone.0080201 24282523 PMC3839934

[B56] Preethirani P. L. Isloor S. Sundareshan S. Nuthanalakshmi V. Deepthikiran K. Sinha A. Y. (2015). Isolation, biochemical and molecular identification, and in-vitro antimicrobial resistance patterns of bacteria isolated from bubaline subclinical mastitis in South India. *PLoS One* 10:e0142717. 10.1371/journal.pone.0142717 26588070 PMC4654528

[B57] Queipo-Ortuño M. I. Colmenero J. D. Macias M. Bravo M. J. Morata P. (2008). Preparation of bacterial DNA template by boiling and effect of immunoglobulin G as an inhibitor in real-time PCR for serum samples from patients with brucellosis. *Clin. Vaccine Immunol.* 15 293–296. 10.1128/CVI.00270-07 18077622 PMC2238042

[B58] Reyes-Jara A. Cordero N. Aguirre J. Troncoso M. Figueroa G. (2016). Antibacterial effect of copper on microorganisms isolated from bovine mastitis. *Front. Microbiol.* 7:626. 10.3389/fmicb.2016.00626 27199953 PMC4848319

[B59] Rovira P. McAllister T. Lakin S. M. Cook S. R. Doster E. Noyes N. R. (2019). Characterization of the microbial resistome in conventional and “raised without antibiotics” beef and dairy production systems. *Front. Microbiol.* 10:1980. 10.3389/fmicb.2019.01980 31555225 PMC6736999

[B60] Schaik W. (2015). The human gut resistome. *Philos. Trans. R. Soc. B* 370:20140087. 10.1098/rstb.2014.0087 25918444 PMC4424436

[B61] Schönborn S. Wente N. Paduch J. H. Krömker V. (2017). In vitro ability of mastitis causing pathogens to form biofilms. *J. Dairy Res.* 84 198–201. 10.1017/S0022029917000218 28524019

[B62] Seth S. Välimäki N. Kaski S. Honkela A. (2014). Exploration and retrieval of whole-metagenome sequencing samples. *Bioinformatics* 30 2471–2479. 10.1093/bioinformatics/btu340 24845653 PMC4230234

[B63] Singh S. Singh S. K. Chowdhury I. Singh R. (2017). Understanding the mechanism of bacterial biofilms resistance to antimicrobial agents. *Open Microbiol. J.* 11:53. 10.2174/1874285801711010053 28553416 PMC5427689

[B64] Su J. Q. An X. L. Li B. Chen Q. L. Gillings M. R. Chen H. (2017). Metagenomics of urban sewage identifies an extensively shared antibiotic resistome in China. *Microbiome* 5:84. 10.1186/s40168-017-0298-y 28724443 PMC5517792

[B65] Thompson-Crispi K. Atalla H. Miglior F. Mallard B. A. (2014). Bovine mastitis: Frontiers in immunogenetics. *Front. Immunol.* 5:493. 10.3389/fimmu.2014.00493 25339959 PMC4188034

[B66] Tiwari S. Jamal S. B. Hassan S. S. Carvalho P. V. S. D. Almeida S. Barh D. (2017). Two-component signal transduction systems of pathogenic bacteria as targets for antimicrobial therapy: an overview. *Front. Microbiol.* 8:1878. 10.3389/fmicb.2017.01878 29067003 PMC5641358

[B67] Tomazi T. de Souza A. F. Heinemann M. B. Dos-Santos M. V. (2018). Molecular characterization and antimicrobial susceptibility pattern of Streptococcus agalactiae isolated from clinical mastitis in dairy cattle. *PLoS One* 13:e0199561. 10.1371/journal.pone.0199561 29928042 PMC6013152

[B68] Vaidya M. Y. McBain A. J. Butler J. A. Banks C. E. Whitehead K. A. (2017). Antimicrobial efficacy and synergy of metal ions against *Enterococcus faecium*, *Klebsiella pneumoniae* and *Acinetobacter baumannii* in planktonic and biofilm phenotypes. *Sci. Rep.* 7:5911. 10.1038/s41598-017-05976-9 28724953 PMC5517536

[B69] Van Boeckel T. P. Brower C. Gilbert M. Grenfell B. T. Levin S. A. Robinson T. P. (2015). Global trends in antimicrobial use in food animals. *Proc. Natl. Acad. Sci. U.S.A.* 112 5649–5654. 10.1073/pnas.1503141112 25792457 PMC4426470

[B70] Weller C. Wu M. A. (2015). A generation-time effect on the rate of molecular evolution in bacteria. *Evolution* 69 643–652. 10.1111/evo.12597 25564727

[B71] Yao H. Shen Z. Wang Y. Deng F. Liu D. Naren G. (2016). Emergence of a potent multidrug efflux pump variant that enhances Campylobacter resistance to multiple antibiotics. *mBio* 7:e01543-16. 10.1128/mBio.01543-16 27651364 PMC5030363

[B72] Zaheer R. Lakin S. M. Polo R. O. Cook S. R. Larney F. J. Morley P. S. (2019). Comparative diversity of microbiomes and Resistomes in beef feedlots, downstream environments and urban sewage influent. *BMC Microbiol.* 19:197. 10.1186/s12866-019-1548-x 31455230 PMC6712873

[B73] Zaheer R. Noyes N. Polo R. O. Cook S. R. Marinier E. Van Domselaar G. (2018). Impact of sequencing depth on the characterization of the microbiome and resistome. *Sci. Rep.* 8:5890. 10.1038/s41598-018-24280-8 29651035 PMC5897366

[B74] Zatakia H. M. Arapov T. D. Meier V. M. Scharf B. E. (2018). Cellular stoichiometry of methyl-accepting chemotaxis proteins in Sinorhizobium meliloti. *J. Bacteriol.* 200 e614–e617. 10.1128/JB.00614-17 29263102 PMC5826028

[B75] Zere T. R. Vakulskas C. A. Leng Y. Pannuri A. Potts A. H. Dias R. (2015). Genomic targets and features of BarA-UvrY (-SirA) signal transduction systems. *PLoS One* 10:e0145035. 10.1371/journal.pone.0145035 26673755 PMC4682653

